# Case report: Different clinical manifestations of the rare Loeffler endocarditis

**DOI:** 10.3389/fcvm.2022.970446

**Published:** 2022-11-29

**Authors:** Yuanyuan Zhao, Peiqing Jiang, Xiangyun Chen, Guihua Yao

**Affiliations:** ^1^Department of Cardiology, Cheeloo College of Medicine, Qilu Hospital (Qingdao), Shandong University, Qingdao, China; ^2^Key Laboratory of Cardiovascular Remodeling and Function Research, Chinese Ministry of Education, Jinan, Shandong, China; ^3^Chinese National Health Commission, Chinese Academy of Medical Sciences, Jinan, Shandong, China; ^4^The State and Shandong Province Joint Key Laboratory of Translational Cardiovascular Medicine, Jinan, Shandong, China; ^5^Department of Cardiology, Qilu Hospital, Cheeloo College of Medicine, Shandong University, Jinan, Shandong, China

**Keywords:** Loeffler endocarditis, eosinophilia, thrombus, corticosteroid, anticoagulant

## Abstract

**Background:**

Loeffler endocarditis is a rare and fatal disease, which is prone to be misdiagnosed, owing to its various clinical manifestations. Consequently, an early identification of Loeffler endocarditis and its effective treatment are crucial steps to be undertaken for good prognosis.

**Case presentation:**

This report describes two cases of Loeffler endocarditis with different etiologies and clinical manifestations. Case 1 was caused by idiopathic eosinophilia and presented with a thrombus involving the tricuspid valve and right ventricular inflow tract (RVIT). The patient suffered from recurrent syncope following activity. After the patient underwent tricuspid valve replacement and thrombectomy, he took oral prednisone and warfarin for 2 years, consequent to which he discontinued both drugs. However, the disease recurred 6 months later, this time manifesting as edema of both legs. Echocardiography showed that a thrombus had reappeared in the RVIT. Thus, oral prednisone and warfarin therapy was readministered. Three months later, the thrombus had dissolved. Low-dose prednisone maintenance therapy was provided long term. Case 2 involved a patient who presented with recurrent fever, tightness in the chest, and asthma, and whose condition could not be confirmed, despite multiple local hospitalizations. In our hospital, echocardiography revealed biventricular apical thrombi. After comprehensive examinations, the final diagnosis was eosinophilic granulomatosis polyangiitis (EGPA) involving multiple organs, including the heart (Loeffler endocarditis), lungs, and kidneys. After administration of corticosteroid, anticoagulant, and immunosuppressive agents along with drugs to improve cardiac function, the patient's symptoms improved significantly.

**Conclusion:**

In Loeffler endocarditis due to idiopathic eosinophilia, long-term corticosteroid use may be required. Diverse and non-specific symptoms cause Loeffler endocarditis to be easily misdiagnosed. So, when a patient shows a persistent elevation of the eosinophil count with non-specific myocardial damage, the possibility of this disease, should always be considered. Furthermore, even when an invasive clinical procedure such as endomyocardial biopsy (EMB) is not available or acceptable, corticosteroids should be administered promptly to bring the eosinophil count back to the normal range, thereby halting the progression of disease and reducing patient mortality.

## Introduction

Loeffler endocarditis, also known as eosinophilic endocarditis, is a severe complication of eosinophilia (1). As this condition manifests as diverse clinical presentations, it is easily misdiagnosed as a cardiac emergency such as acute myocarditis or acute myocardial infarction. If left untreated, the patients may develop potentially fatal complications such as cardioembolism, arrhythmia, or acute heart failure (2). Consequently, early identification and effective treatment are crucial for improving the prognosis. However, no consensus guidelines are available for managing Loeffler endocarditis (3), because the management treatment of each patient is individualized and relates to (1) an acute treatment, (2) a symptomatic cardiac treatment based on diverse manifestations (anti-arrhythmics, pressure support, ejection fraction support (EF support), thrombus targeting, prophylaxis, etc.), and (3) finally a thorough diagnostic workup to identify the cause of eosinophilia (idiopathic, primary, or secondary). This report presents in a detailed manner how we had used these three components in the treatment of our two patients. We hope that it provides valuable information for the early recognition and appropriate treatment of this rare and fatal disease.

## Case description

### Case 1

**Figure 1:**
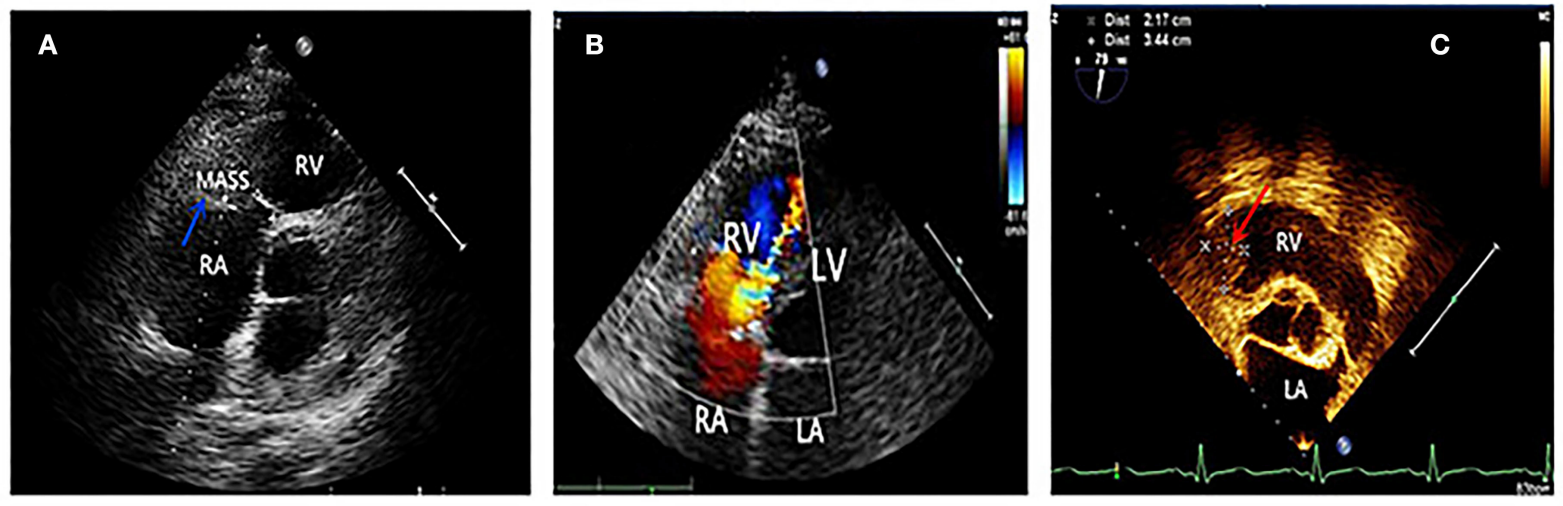
**(A)** Blue arrow showed a mass in the right ventricle, which was later confirmed thrombus by surgery. **(B)** Four chamber view showed the mosaic blood flowing through the stenotic right ventricular inflow tract. **(C)** Two and a half years after tricuspid valve replacement and thrombectomy, a recurrence of thrombus (indicated by red arrow) was seen in the right ventricular inflow tract by transesophageal echocardiography.

A 61-year-old man was admitted to our hospital on May 20, 2016, due to syncope after activity. One month before that incident, he had experienced tightness in his chest and dizziness, with both symptoms showing reprieve following a short rest (3–5 min). A few days later, he felt his condition was progressively worsening with each passing day. He occasionally had sudden syncope during activity, but it then revived on its own without any intervention. On admission to our hospital, his blood pressure was 110/70 mmHg. An electrocardiogram showed flat T waves in leads II, III, and arteriovenous fistula (AVF). Routine blood work showed a white blood cell (WBC) count of 11.4 × 10^9^/L (reference, 4–10 × 10^9^/L), eosinophil count of 3.1 × 10^9^/L (reference, 0.02–0.52 × 10^9^/L) with a percentage of eosinophils at 38% (reference, 0.4–8.0%), a hemoglobin level of 135 g/L (reference, 120–160 g/L), and a platelet count of 146 × 10^9^/L (reference, 100–300 × 10^9^/L). Moreover, the level of cardiac troponin I (cTnI) was 0.05 ng/mL (nanograms/milliliter) (reference, <0.2 ng/mL), and the level of B-type natriuretic peptide (BNP) was 441 pg/mL (pictograms/milliliter) (reference, <100 pg/mL). Cardiac computed tomography (CT) showed right atrial (RA) enlargement, small right ventricle (RV), and small pericardial effusion. Echocardiography showed a mass (Figure 1A) in the right ventricle (RV) involving the right ventricular inflow tract (RVIT), tricuspid annulus, chordae tendineae, and papillary muscle, resulting in RVIT stenosis (Figure 1B) and restriction of the motion of the tricuspid leaflet and chordae. Left ventricular ejection fraction (LVEF) was 66%. Contrast echocardiography showed a mass in the right ventricle and no blood perfusion within it. The patient did not show any history of hypertension, cerebrovascular disease, coronary heart disease, heart failure, arrhythmia, food and drug allergies, infectious diseases, or parasitic infection. Parasite and leukemic fusion gene [Fip1-like 1/platelet-derived growth factor receptor-alpha (FIP1/PDGFR)] tests were all negative. Eosinophilia and Loeffler endocarditis with thrombus in the RV was considered a probable diagnosis.

### Case 2

A 58-year-old man presented with chest tightness, cough, and asthma without fever in late October 2020. The patient took aminophylline and inhaled budesonide, but without obvious effect. His condition began to worsen gradually and he was admitted to a local hospital on 4 November, 2020. Electrocardiogram showed an elevated ST segment in leads II, III, and arteriovenous fistula (AVF). Echocardiography showed segmental wall motion abnormalities, and the LVEF was decreased (36%). Laboratory examinations showed elevated cardiac troponin I (cTnI) and N-terminal pro-B-type natriuretic peptide (NT-proBNP) levels. Thus, the patient was transferred to a cardiology care unit (CCU) for acute inferior myocardial infarction and heart failure. Coronary computed tomography (CT) angiography results showed only mild stenosis in the coronary arteries. The patient's symptoms improved insignificantly after the administration of medications that improved cardiac function. During hospitalization, he had fever with temperature of 39.5°C. Blood work showed a white blood cell (WBC) count of 9.7 × 10^9^/L, hemoglobin level of 117 g/L, platelet count of 186 × 10^9^/L, and high levels (2.2 × 10^9^/L) and percentage (22.8%) of eosinophils. As the patient presented with cough and asthma, he was administered anti-infective and antipyretic treatment. Four days later, the patient's body temperature decreased to normal, and the fever symptoms were relieved. He was discharged from the hospital on 19 November, 2020. Two weeks later, the symptoms including fever, cough, and asthma recurred. Upon revisiting the local hospital, the patient's chest CT showed bronchiectasis with infection. He was administered antibiotics for 7 days but with poor effect and had fever with a temperature of 38°C.

**Figure 2:**
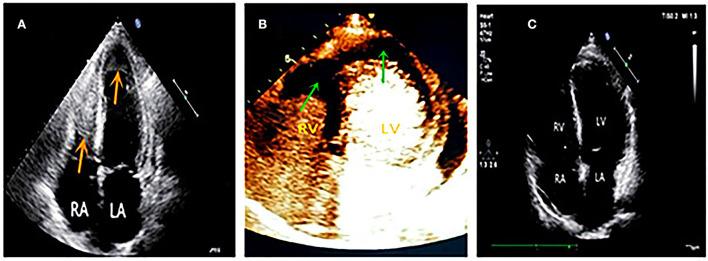
**(A)** Four chamber view showed masses in both of the ventricular apex (red arrows). **(B)** Contrast echocardiography showed no perfusion in both apical masses (green arrows), which suggested thrombi. **(C)** Follow-up at six months later, both ventricular apical thrombi were disappeared.

With no significant relief from symptoms, he revisited our hospital and was admitted to the respiratory department with bronchiectasis. Physical examinations revealed a body temperature of 37.6°C, pulse rate of 111 times/min, respiration rate of 20 breaths/min, and blood pressure of 130/80 mmHg. The laboratory examination showed the following test results: eosinophil count 3.36 × 10^9^/L, percentage of eosinophils 32.10%, cTnI level of 1.032 ng/mL, and BNP level of 1,073 pg/mL. The patient had 1-year history of bronchial asthma and allergic rhinitis. He had a 30-year smoking history, smoking approximately 7 cigarettes per day and quitting smoking for 1 year. The patient did not show any history of hypertension, coronary heart disease, diabetes mellitus, food or drug allergies, infectious diseases, or parasitic infections. Echocardiography showed an enlarged heart and decreased LVEF (47%). It also showed that there were masses in both ventricular apexes with a small amount of pericardial effusion (Figure 2A). Contrast echocardiography showed no perfusion in either of the ventricular apical masses (Figure 2B). Hence, we considered the masses to be thrombi.

## Diagnostic assessment and therapeutic interventions

### Case 1

Eosinophilia and Loeffler endocarditis with thrombus in the RV was considered a probable diagnosis. The patient was administered low-molecular-weight heparin (subcutaneous injection, 5,000 IU (international units), twice daily) and oral prednisone (60 mg, once daily). Nine days later, a routine blood work showed that eosinophils had decreased to within the normal range (0.37 × 10^9^/L); however, frequent syncope persisted. The patient then underwent thrombectomy and tricuspid valve replacement. Pathological results showed an RV thrombus, endocardial fibroplasia, and eosinophilic infiltration. The symptoms relieved significantly after the operation, and he was discharged with oral prednisone (60 mg, once daily) and warfarin (3.75 mg, once daily). Six months later, prednisone was gradually tapered down to 10 mg daily. The patient continued to feel well for 2 years following surgery when he stopped taking prednisone and warfarin on his own. However, 6 months later, the patient experienced edema in both legs and was readmitted. Blood work showed elevated eosinophils once again (5.13 × 10^9^/L). Echocardiography revealed a hypoechoic mass attached to the RVIT (Figure 1C), and contrast echocardiography showed no perfusion in the mass. Thus, the patient was diagnosed to have a recurrence of thrombus . He was once again prescribed oral prednisone (60 mg, once daily) and warfarin (4.5 mg, once daily). At the 3-month follow-up, no edema was noticed in the patient's legs. The eosinophils had restored to normal conditions, and the RV thrombus had dissolved. Thus, the medication was changed to long-term low-dose prednisone (10 mg, once daily) maintenance therapy. The patient remained in good health at later follow-up visits.

### Case 2

A probable diagnosis of Loeffler endocarditis was considered. The patient was advised to undergo an endomyocardial biopsy but he refused invasive testing. He also refused to undergo cardiac magnetic resonance (CMR) due to noise intolerance. A bone marrow puncture showed eosinophilia. Genetic FIP1L1/PDGFRA, platelet-derived growth factor receptor-beta (PDGFRB), FGFR1/d8z2 (8p11), and parasite testing were negative. Other abnormal examination results included immunoglobulin G (IgG) 24.9 g/L↑, immunoglobulin E (IgE) 1,890 IU /mL↑, and urinary microalbumin 122.74 mg/L↑. Combined with clinical symptoms and examinations, the final diagnosis was eosinophilic granulomatosis polyangiitis (EGPA) involving multiple organs, including the heart (Loeffler endocarditis), lungs, kidneys, and nasal sinuses. This diagnosis was made according to the Classification Criteria of the American College of Rheumatology for eosinophilic granulomatosis polyangiitis (EGPA) (4). The therapeutic medications included prednisone and mycophenolate mofetil for anti-immunotherapy, warfarin for anticoagulation, sacubitril valsartan, metoprolol, spironolactone, and dapagliflozin for heart failure. The patient's temperature returned to normal, and symptoms of tightness in chest, shortness of breath, and asthma alleviated substantially. During the regular outpatient follow-up visits, eosinophil count, IgG, and IgE levels returned to the normal range, electrocardiogram returned to a normal pattern, LVEF was improved to above 50%, and mural thrombi in both ventricles disappeared in echocardiography (Figure 2C). The patient's exercise tolerance improved significantly and he reported the ability to play basketball for up to an hour. Prednisone and mycophenolate mofetil were gradually tapered down to a maintenance dose. Because the patient had no uncomfortable symptoms and was in a good condition, he requested discontinuation of the medications for heart failure. A recent follow-up reported normal immunoglobulin levels, normal cardiac function, and no mural thrombus in the heart.

## Discussion

Eosinophilia is defined as a persistent peripheral blood eosinophil count ≥ 1.5 × 10^9^/L with an extensive or local eosinophilic infiltration (5), which involves multiple organs, including the heart, lungs, kidneys, central nervous system, and skin. Cardiac involvement is the most serious complication of this condition. Its incidence is over 50% and is a major cause of morbidity and mortality (6). Eosinophilia can be classified as idiopathic, primary, and secondary according to the etiology (7). The etiologies of primary eosinophilia include stem cells, myeloid or eosinophilic neoplasm (PDGFRA, PDGFRB, FGFR1), and T-cell lymphoma. The etiologies of secondary eosinophilia include parasitic infections, allergic diseases (bronchial asthma, drug allergies), certain infectious diseases (tuberculosis, AIDS), and hematologic diseases (8). However, there is no apparent etiology in idiopathic eosinophilia.

Loeffler endocarditis refers to eosinophilia that involves the endocardium, with the first case having been reported by Loeffler in 1936 (9). According to its pathological characteristics, Loeffler endocarditis can be divided into three phases: (1) the necrotic phase: eosinophils infiltrate the myocardium, and inflammation leads to myocardial damage and necrosis; (2) the thrombotic phase: as myocardial inflammation subsides, the heart cavity subsequently forms a mural thrombus; (3) the fibrotic phase: eosinophils disappear, and the primary characteristic is extensive endocardial fibrous proliferation. In the third phase, the ventricular diastolic function is the main pathophysiological change, often manifested as recalcitrant chronic heart failure (10). Once it progresses to the fibrotic stage, the survival rate is only 35%−50%, even after 2 years of active treatment (11). As the disease progresses, serious outcomes, such as heart failure, arrhythmia, and embolic events, manifest (12). In the early stages, it is easy to misdiagnose this condition due to diverse clinical manifestations (13).

Endomyocardial biopsy is the gold standard for diagnosing Loeffler endocarditis (14); however, no clinical guidelines for managing Loeffler endocarditis are currently in vogue, and treatment expertise is mostly derived from a handful of previous case reports. The management of Loeffler endocarditis requires consideration of the etiology of eosinophilia, the stage of progress, and the involvement of organs (3). The treatment goals should include the following: (1) eosinophils being reduced to the normal range to avoid cardiac damage (eosinophils can release toxic substances), and (2) treatment of the underlying etiology and complications (heart failure, arrhythmia, thrombus). Primary eosinophilia, especially in patients with FIP1L1-PDGFRA-positive, is highly sensitive to imatinib (15, 16). Imatinib is the first choice for treatment. The Loeffler endocarditis originates from idiopathic and secondary eosinophilia, corticosteroids are usually the primary medication (17). In addition, it is important to save samples for clonality (genetic, flow cytometry) before corticosteroids' initiation, if possible, because corticosteroids often have a very rapid effect, blurring the diagnostic clues.

In our report, Case 1, with idiopathic eosinophilia, had only cardiac involvement. A thrombus caused stenosis of the tricuspid valve and RVIT, leading to decreased cardiac output and frequent syncope. After thrombectomy and tricuspid valve replacement and administration of a corticosteroid and an anticoagulant, the patient felt remarkably better. The current literature does not contain exact guidelines for when to cease corticosteroid administration in idiopathic eosinophilia; however, from Case 1 in this study, we observed that when the patient stopped taking medications after several months, the eosinophil level increased and the thrombus reappeared. This indicated that if the etiology of eosinophilia was unknown, discontinuation of corticosteroids may lead to recurrence of the disease. Thus, corticosteroids may need to be taken long term in idiopathic eosinophilia.

Case 2 was of secondary eosinophilia resulting from an autoimmune disease involving multiple organs and leading to diverse clinical manifestations. At the initial visit, the patient from Case 2 was in the first stage of Loeffler endocarditis, showing non-specific symptoms such as myocardial injury and heart failure. In fact, eosinophils were already high at this stage. However, because of the rarity of Loeffler endocarditis and the lack of awareness of this disease by many physicians, it is prone to be misdiagnosed. In our hospital, echocardiography showed disease progression to the thrombotic phase, with contrast echocardiography playing an important role in the differential diagnosis of the thrombus.

Although the gold standard of diagnosis for Loeffler endocarditis is an endomyocardial biopsy, it can sometimes turn out to be a difficult diagnostic procedure. Because it is an invasive and risky examination, it may also be rejected outright by the patient, as it occurred in Case 2. Moreover, not all hospitals possess the necessary infrastructure, conditions, or technology to perform an endomyocardial biopsy. However, as Loeffler endocarditis is fatal, and where endomyocardial biopsy is not available, corticosteroids should be administered early to patients who present with persistent elevation of eosinophils and non-specific heart damage. Timely treatment alone can reduce the damage of eosinophils to the heart, prevent disease progression, and reduce patient mortality.

Prolonged use of corticosteroids gives rise to an increased risk of osteoporosis and other long-term complications. If the disease is effectively alleviated without recurrence, the corticosteroids should then be tapered to a minimum maintenance dose only. Calcium and vitamin D supplementation can be administered, according to guidelines. However, it is pertinent to conduct a careful diagnostic workup, at diagnosis or at recurrence after terminating steroids, to decide the glucocorticoid-sparing treatments for idiopathic, primary, or secondary eosinophilia with alternative immunosuppressive or cytoreductive agents (6, 7, 15, 16).

In addition, multidisciplinary collaboration is essential for patient management especially because of the different etiologies of Loeffler endocarditis, the diverse clinical presentations, and the possibility of involving multiple organs. All these different spheres of activity have to work in close unison toward effective patient care. This is exactly what happened in Case 2. Through a multidisciplinary collaboration including those of Departments of Cardiology, Respiratory, Hematology, and Rheumatology, the patient was correctly diagnosed as having EGPA and was given an effective therapy with good clinical outcomes.

Despite the prognoses of the two patients being good, our study still has certain limitations. First, patient of Case 2 was in poor health condition when he first visited our hospital. He refused to follow the doctors' suggestions to undergo endomyocardial biopsy and CMR. Therefore, it was unfortunate that we could not obtain the endocardial pathology results of his endocardium. CMR is yet another important non-invasive imaging tool, besides echocardiography, for evaluating tissue properties and detecting thrombus in Loeffler endocarditis (18). Hence, the use of comprehensive multimodality imaging, inclusive of echocardiography, contrast echocardiography, and CMR, should be considered in the future for the evaluation of Loeffler endocarditis, if available. Overall, the two patients are presently in good condition and are satisfied with our diagnosis and treatment.

## Conclusion

When the etiology of eosinophilia remains unknown, eosinophils continue to reelevate if corticosteroids are discontinued. This suggests that corticosteroids should be used long term to prevent the recurrence of idiopathic eosinophilia. In its initial stage, Loeffler endocarditis is prone to be misdiagnosed due to its diverse and unspecific symptoms. When the patients show persistent eosinophil elevation with non-specific myocardial damage, the possibility of this disease, must always be considered. Even if an endomyocardial biopsy is unavailable, corticosteroids should be administered promptly to reduce eosinophil levels to normal, thereby halting the progression of disease and reducing patient mortality.

## Data availability statement

The original contributions presented in the study are included in the article/Supplementary material, further inquiries can be directed to the corresponding author/s.

## Ethics statement

The studies involving human participants were reviewed and approved by Medical Ethics Committee of Qilu Hospital of Shandong University (Qingdao). The patients/participants provided their written informed consent to participate in this study. Written informed consent was obtained from the individual(s) for the publication of any potentially identifiable images or data included in this article.

## Author contributions

YZ initially wrote the manuscript. PJ and XC collected the data. GY reviewed and revised the manuscript. All authors contributed to the diagnosis and treatment of the patients, read the manuscript, and agreed to publication.

## Funding

This study was supported by the National Natural Science Foundation of China (81671703), the Qingdao Key Health Discipline Development Fund (3311000000073), the People's Livelihood Science and Technology Project of Qingdao (18-6-1-62-nsh), and the Qilu Hospital Fund (QDKY2017QN08).

## Conflict of interest

The authors declare that the research was conducted in the absence of any commercial or financial relationships that could be construed as a potential conflict of interest.

## Publisher's note

All claims expressed in this article are solely those of the authors and do not necessarily represent those of their affiliated organizations, or those of the publisher, the editors and the reviewers. Any product that may be evaluated in this article, or claim that may be made by its manufacturer, is not guaranteed or endorsed by the publisher.

## Abbreviations

EGPA: Eosinophilic granulomatosis polyangiitis
LVEF: Left ventricular ejection fraction
RVIT: Right ventricular inflow tract
LA: Left atrial
RA: Right atrial
LV: Left ventricle
RV: Right ventricle
cTnI: Cardiac troponin I
NT-proBNP: N-terminal pro-B-type natriuretic peptide
BNP: B-type natriuretic peptide.
