# Supplementary figures and images for: Case report: Different clinical manifestations of the rare Loeffler endocarditis
Source: Front Cardiovasc Med. 2022 Nov 29;9:970446. doi: 10.3389/fcvm.2022.970446 (PMC9745302; doi:10.3389/fcvm.2022.970446)

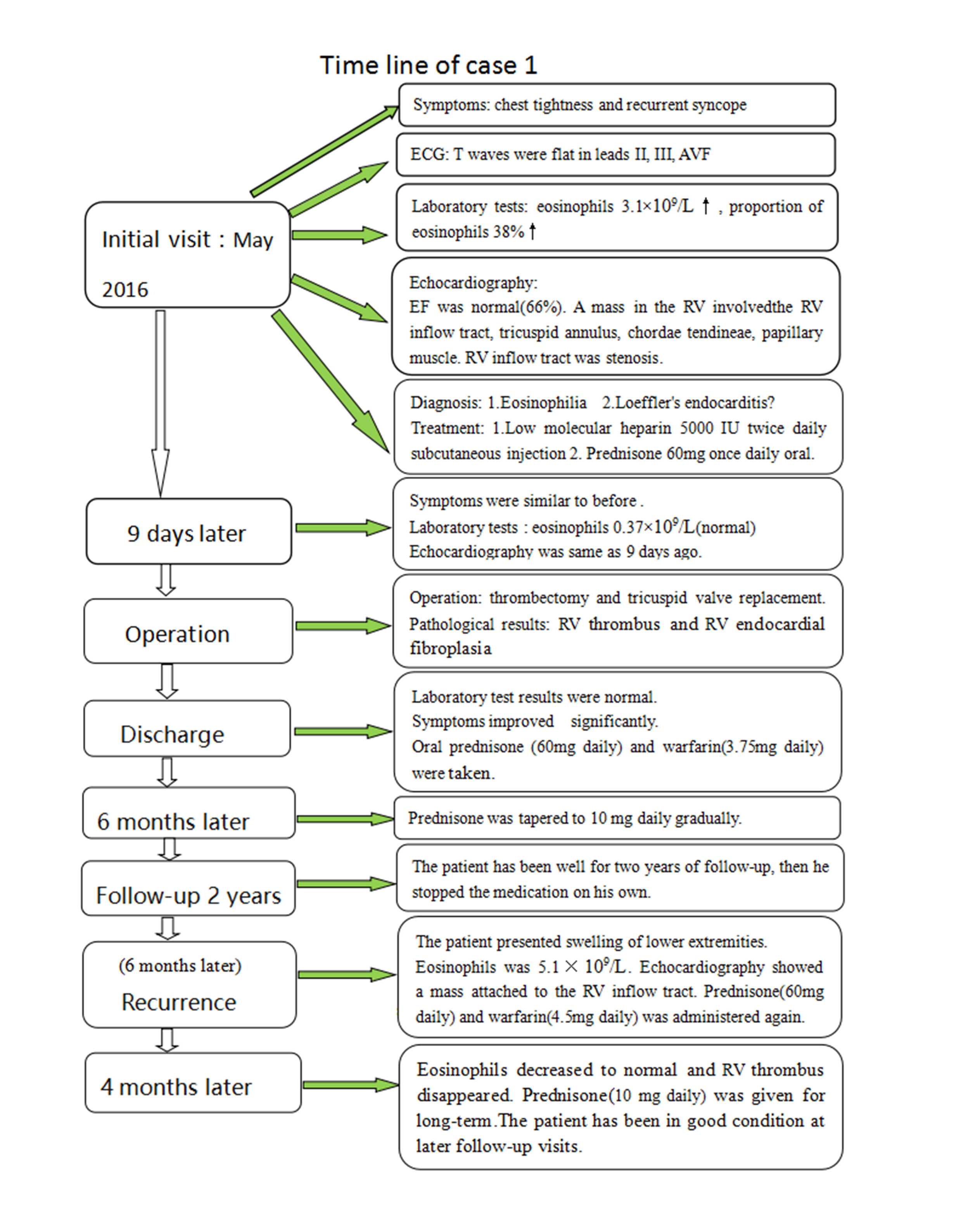

Supplement: Supplementary file 1 [file Image_1.TIF]

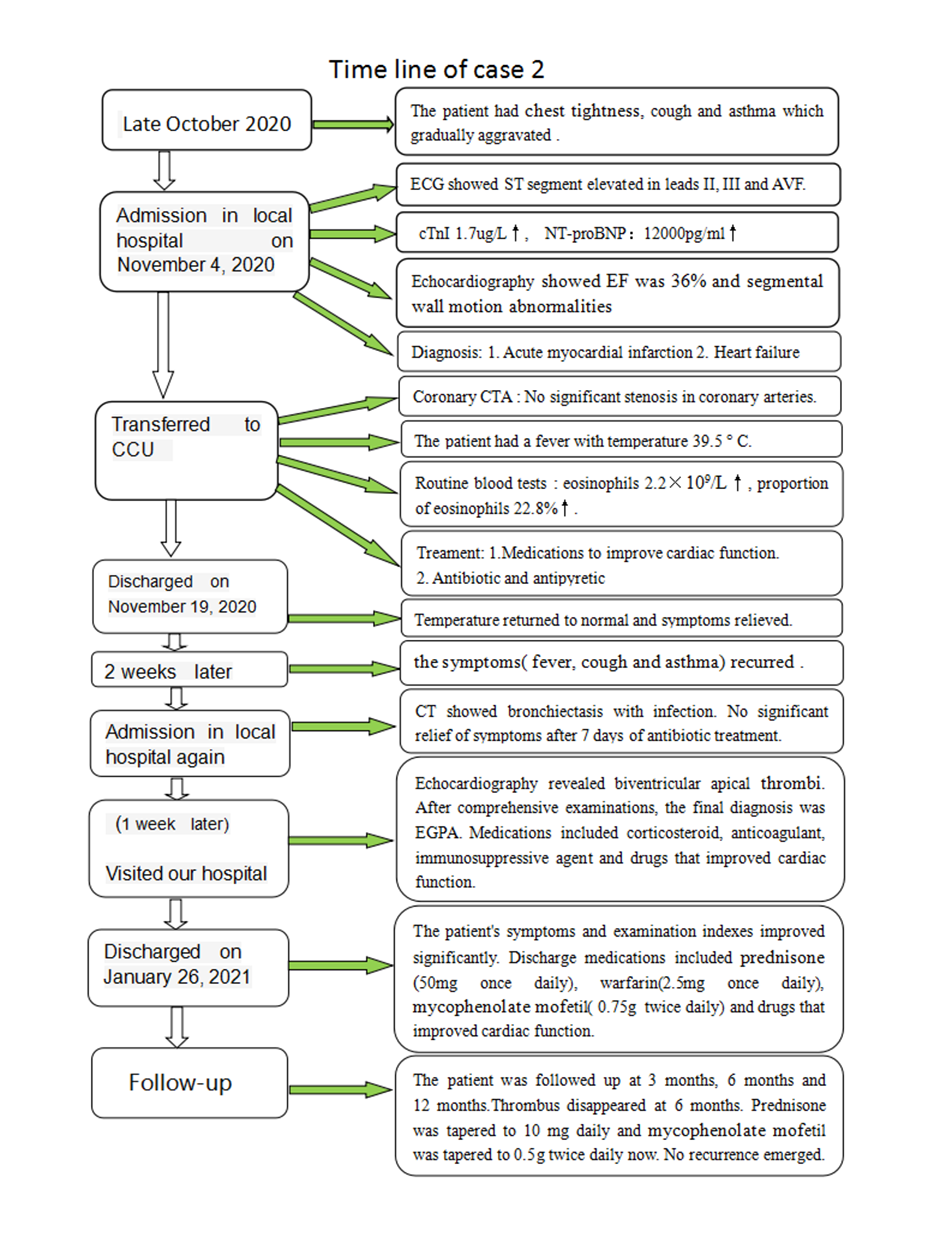

Supplement: Supplementary file 2 [file Image_2.TIF]
